# Unveiling Leukocyte Extracellular Traps in Inflammatory Responses of the Central Nervous System

**DOI:** 10.3389/fimmu.2022.915392

**Published:** 2022-07-01

**Authors:** Francesca Colciaghi, Massimo Costanza

**Affiliations:** ^1^ Epilepsy Unit, Fondazione IRCCS Istituto Neurologico Carlo Besta, Milan, Italy; ^2^ Molecular Neuro-Oncology Unit, Fondazione IRCCS Istituto Neurologico Carlo Besta, Milan, Italy

**Keywords:** extracellular DNA, ETosis, sterile inflammation, neuroinflammation, central nervous system

## Abstract

Over the past nearly two decades, increasing evidence has uncovered how immune cells can actively extrude genetic material to entrap invading pathogens or convey sterile inflammatory signals that contribute to shaping immune responses. Originally identified in neutrophils, the release of decondensed chromatin fibers decorated with antimicrobial proteins, called extracellular traps (ETs), has been recognized as a specific form of programmed inflammatory cell death, which is now known to occur in several other leukocytes. Subsequent reports have shown that self-DNA can be extruded from immune cells even in the absence of cell death phenomena. More recent data suggest that ETs formation could exacerbate neuroinflammation in several disorders of the central nervous system (CNS). This review article provides an overview of the varied types, sources, and potential functions of extracellular DNA released by immune cells. Key evidence suggesting the involvement of ETs in neurodegenerative, traumatic, autoimmune, and oncological disorders of the CNS will be discussed, outlining ongoing challenges and drawing potentially novel lines of investigation.

## Introduction

In recent years a large body of literature has provided compelling evidence that self-DNA, aside from carrying genetic information, can trigger multiple innate immune pathways that contribute to inflammatory responses (1). This notion is dramatically exemplified in polytrauma pathology, when accidental cell lysis results in the leakage of large quantities of mitochondrial (mt) formyl-peptides and mtDNA into the body fluids, promoting massive neutrophil activation and a septic shock-like syndrome (2). Because of its ancestral prokaryotic origin, mtDNA has analogies with bacterial DNA as a circular structure and the presence of a large number of unmethylated CpG sequences (3), which bind toll-like receptor (TLR)-9 (4) and potently promote inflammation (5). Later it was shown that mtDNA also activates the NOD-like receptor (NLR) family pyrin domain containing 3 (NLRP3) inflammasome (6) and the cyclic GMP-AMP synthase (cGAS)-STING cytosolic DNA sensing pathway (7).

While this was only an extreme example of the inflammatory potential of DNA and represents a random and uncontrolled event, the release of mitochondrial and nuclear DNA complexed with proteins by immune cells has increasingly emerged as a novel inflammatory process (8). In 2004, Zychlinsky’s group demonstrated that neutrophils activated with phorbol esters extrude chromatin fibers decorated with anti-microbial proteins that entrap and kill bacteria *in vitro*, thus termed neutrophil extracellular traps (NETs) (9). Since then, a considerable amount of work has been done to investigate the composition of NETs, the extracellular cues, and the intracellular pathways leading to NETs formation and their role in physiological and pathological immune responses. It is now known that extracellular traps (ETs) release occurs in several leukocytes in addition to neutrophils (10) and is mostly considered an inflammatory cell death program called ETosis (11). A limited number of reports have also raised the possibility of a vital DNA release from both innate and adaptive immune cells (12).

The spectrum of diseases implicating extracellular DNA threads derived from immune cells has progressively expanded from defense against microbes to a wide plethora of other inflammatory conditions, including neurological disorders, where they can convey sterile inflammatory signals that exacerbate immune pathology (13).

This review article will provide a critical and up-to-date overview of the various types of extracellular DNA, the immune cells from which they originate, their composition, and their proposed function. Next, we will focus specifically on the involvement of leukocyte-derived extracellular DNA fibers in the most common noninfectious pathologies of the central nervous system (CNS), discussing major advances and unanswered questions in the field.

## Extracellular DNA From Leukocytes: Multiple Blends and Modalities of Extrusion

### Suicidal NETosis: Features and Proposed Effector Functions

When Brinkmann and colleagues first documented in 2004 that neutrophils, the most abundant innate immune cells, release NETs following stimulation with phorbol myristate acetate (PMA), interleukin (IL)-8, or lipopolysaccharide (LPS), they concluded that this phenomenon did not appear to be the result of cell membrane rupture, but they did not rule out an initial phase of programmed cell death (9). This second option has been adopted as the main biological framework for interpreting and studying this process since the same group showed in 2007 that a four-hour stimulation of neutrophils with PMA sequentially induces nuclear envelope fragmentation, chromatin decondensation, and cytoplasmic granule disintegration, with the consequent mixing of nuclear, cytoplasmic, and granular components to form NETs (14). It has been also demonstrated that neither apoptotic nor necrosis triggers, such as an anti-Fas antibody (Ab) or bacterial toxins, respectively, elicit NETs formation, suggesting that NETs are the consequence of a novel cell death program, later named NETosis by Steinberg and Grinstein (15).

The formation of NETs is prompted by a wide plethora of stimuli such as bacteria, fungi, parasites, viruses, complement factors, platelets, immune complexes, crystals, and cytokines that activate immune receptors such as TLRs, Dectin-2, FcR, and others. An extensive description of inducers, receptors, and intracellular mediators of NETosis has been recently provided (10, 16) and is out of the scope of this review. Electron microscopy imaging of PMA-induced NETs has revealed the presence of DNA threads with highly different diameters ranging from 15 nm up to 50 nm (9). Mass-spectrometry analysis of these DNA fibers has identified at least 24 different associated-proteins that include leukocyte elastase, calprotectin, cathepsin G, defensins, myeloperoxidase (MPO), histones, actin, and α-actinin (17). Another work has characterized 33 different proteins in NETs elicited by several strains of *P. aeruginosa*, showing a general overlap with PMA-derived NETs as they included histones and granule or cytoplasmic proteins such as elastase and α-actinin, respectively (18).

An important aspect not to neglect is that in neutrophils stimulated with PMA, which is maybe the most potent inducer of NETosis, NETs-DNA is approximately 10-30% of the average genomic DNA content, indicating that only a fraction of neutrophils eventually undergoes NETosis (14). Why this occurs is still debated and is complicated by the lack of specific markers identifying potentially different neutrophil subsets. A possible interrelationship between NETosis and aging has been drawn by studies demonstrating that aged neutrophils are more prone to form NETs either spontaneously or after exposure to triggers such as LPS, PMA, or ionomycin (19, 20).

The intracellular steps leading to NETosis have been characterized more in detail upon exposure to PMA, which, through protein kinase C (PKC) stimulation, induces nicotinamide adenine dinucleotide phosphate (NADPH) oxidase to produce reactive oxygen species (ROS) (14). Hydrogen peroxide elicits (MPO)-dependent activation of neutrophil elastase (NE), which translocates from azurophilic granules to the cytoplasm where it cleaves actin cytoskeleton to inhibit phagocytosis (21). Subsequently, NE translocates to the nucleus where it starts the proteolytic cleavage of histone H4, partially dismantling chromatin structure, followed by MPO that contributes to chromatin disassembly independently of its enzymatic activity (22). However, the formation of NETs does not always rely on ROS production and NADPH oxidase activity, as shown in response to infection with certain bacteria (23) or parasites (24). Another key intracellular player of NETosis is the enzyme peptidyl-arginine deiminase (PAD)4. PADs are a family of highly conserved proteins (PAD1-PAD4 and PAD6) that mediate protein citrullination. PAD4 has a nuclear localization sequence and is activated by hydrogen peroxide, calcium, and PKCζ (16, 25). PAD4 promotes histone citrullination, i.e. the substitution of arginine residues with the amino acid citrulline, obtained by removing an ammonium positively-charged group (NH4^+^) from arginine. The consequent loosening of the interactions between positively-charged histones and the negatively-charged DNA results in chromatin decondensation, a pre-requisite for NETs extrusion (26). Based on these findings, citrullinated histone H3 (CitH3) is used routinely as a marker for NETs. *Padi4* ^-/-^ neutrophils are unable to form NETs in response to many stimuli such as bacteria (26, 27), LPS (28, 29), tumor necrosis factor (TNF) (28), and nicotine (30), but PAD4 is dispensable for cholesterol crystals induced-NETosis (31).

Potential overlap between NETosis and necroptosis, another inflammatory cell death program, has been suggested by Desai and colleagues, showing that neutrophils from receptor-interacting protein kinase (*Ripk)3* ^-/-^ mice or treated with inhibitors of RIPK-1 or the mixed lineage kinase domain-like (MLKL) are unable to release NETs elicited by monosodium urate crystals or PMA (32). In the same journal issue, another group has provided opposite results, i.e. dispensability of the necroptosis kinases MLKL and RIPK3 for NETs release (33). A possible explanation of these differences lies in the diverse incubation times with the NETotic stimulus used in these two works. Indeed neutrophils were treated with PMA for 15-45 minutes in the latter report and for 120 minutes in the work by Desai and colleagues. More recently, one group has performed an exhaustive characterization of the interplay between NETosis and necroptosis, showing that necroptotic neutrophils release NETs, as revealed by double-strand DNA, CitH3, and elastase staining (34). However, although RIPK-1, RIPK-3, and MLKL enzymes were necessary for NETs formation from necroptotic neutrophils, they were dispensable for PMA-induced NETs (34). Also, the induction of necroptotic NETs requires ROS production but is independent of NADPH oxidase (34). The analysis of PAD4 involvement in necroptotic NETs induction has revealed that *Padi4* ^-/-^ neutrophils are significantly more susceptible to necroptosis. Nonetheless, necroptotic *Padi4* ^-/-^ neutrophils display chromatin decondensation similar to wild-type counterparts, but form reduced NETs, suggesting that PAD4 contributes to chromatin extrusion in this process (34). Overall, these data indicate that, although necroptosis and NETosis share some features and intracellular mediators, they should be accounted for as different cell death programs. Interestingly, gasdermin D, a pore-forming protein involved in pyroptotic cell death, promotes NETosis (35).

Given that one of the main effector functions of neutrophils is to kill invading pathogens, since their initial discovery (9), a substantial number of studies has investigated the contribution of NETs to host defense, proposing that their formation is a microbicidal mechanism deployed by neutrophils in addition or substitution to phagocytosis, as when neutrophils have to face with large microbes (reviewed in (16)). The defensive activity of NETs consists of two different and concomitant mechanisms intrinsically suggested by their structure: the first one is the entrapping of pathogens within strands of DNA, and the second is pathogen exposition to antimicrobial proteins at high concentrations. In the face of a large body of literature supporting the microbicidal properties of NETs, some skepticism has emerged more recently on the actual involvement of this effector mechanism in host defense (25, 36). Indeed, it has been suggested that pathogens are not killed by NETs, as an additional incubation step with DNase *in vitro* was shown to release microbes alive in the culture medium, indicating that NETs might entrap rather than destroy pathogens (37). Furthermore, DNase treatment, often utilized *in vitro* and *in vivo* for demonstrating NETs contribution to host protection, might underestimate the direct effect of DNase on bacterial biofilms, which include microbial DNA, and promote their survival (38). Some pathogens also digest NETs, evading their containment with consequent dissemination into the systemic circulation, or even incorporate NETs into their biofilms (39). Therefore, according to some authors, the contribution of NETs might be more relevant in non-infectious disorders (36, 39, 40), where NETs might provide an unwarranted extra-source of damage-associated molecular patterns (DAMPs) that fuel sterile inflammation, as is the case in several neurological disorders (13).

### Vital Release of Extracellular DNA From Neutrophils

Suicidal NETosis is not the sole modality through which neutrophils release extracellular DNA. An alternative way started to be delineated in 2004 by Yousefi and coworkers, demonstrating that mature neutrophils primed with interferon (IFN)-α, IFN-γ, or granulocyte-macrophage colony-stimulating factor (GM-CSF) and then treated with complement factor 5a (C5a) extrude ETs positive for MPO (41). Subsequent work by the same group has found that neutrophils activated with GM-CSF followed by C5a or LPS - a TLR-4 activator - release NETs positive for elastase, MPO, and MitoSOX Red, a mitochondria-targeted probe emitting fluorescence when oxidized by ROS. This process occurs in a NADPH-dependent fashion but without apparent phenomena of cell death (42). PCR analysis of the DNA extracted from the supernatants of cultured neutrophils revealed the presence of mitochondrial but not nuclear DNA (42). Of note, in this setting neutrophils have been cultured for 35 minutes, while suicidal NETs are generally obtained after 2-4 hours of stimulation. The effector functions of these mtDNA extrusions were not clarified in this work.

A few years later Caielli and colleagues have shown that neutrophils from healthy donors cultured for short time *in vitro* spontaneously extrude mitochondrial nucleoids in the supernatants, namely mtDNA complexed with transcription factor A mitochondria (TFAM), a protein involved in mtDNA compaction and maintenance (43). According to the authors’ hypothesis, this is due to a constitutive defect in neutrophil mitophagy, which eliminates damaged mitochondria based on the oxidative status of mtDNA by two different mechanisms. In the absence of oxidized mtDNA, mitochondrial components, including nucleoids of mtDNA/TFAM, are released into the extracellular space through exocytosis, while mtDNA undergoing oxidation is dissociated from TFAM and routed to lysosomes for degradation (43). This suboptimal disposal of damaged mitochondria in neutrophils exhibits its limitations under inflammatory conditions. Indeed, activation of TLR-7 by anti-Smith (Sm)/ribonucleoprotein (RNP) autoantibodies in neutrophils from healthy donors previously exposed to IFN-α, or from patients with systemic lupus erythematosus (SLE), causes the extrusion of oxidized mitochondrial nucleoids into the extracellular space, instead of being directed to the lysosomes (43). These mitochondrial nucleoids stimulate IFN production by plasmacytoid dendritic cells, through the activation of TLR-9 and receptor for advanced glycation endproducts (RAGE) by oxidized mtDNA and TFAM, respectively (43). The authors also clarify that these mtDNA/TFAM extrusions are not the result of suicidal NETosis or apoptosis, and their production is dependent on mtROS (43). Whether these DNA fibers are associated with proteins classically described in NETs, such as elastase and MPO, was not investigated in this study.

Another study has obtained that neutrophils stimulated with RNP-containing immune complexes release extracellular threads enriched in mtDNA in a mtROS-dependent way, which authors defined NETs (44). In response to immune complexes, mitochondria appeared hyperpolarized and translocated to the cell membrane (44). In line with the work by Caielli et al., even these extrusions are enriched in oxidized mtDNA and are highly pro-inflammatory. Indeed, treatment of peripheral blood mononuclear cells from healthy donors with these NETs results in a markedly increased expression of pro-inflammatory genes such as *IFNB1*, *TNF*, and *IL6* in a STING-dependent manner (44). Moreover, low-density gradient neutrophils, a cell subset identified in SLE patients (45), spontaneously release NETs enriched in oxidized mtDNA compared to NETs from normal-density gradient neutrophils of healthy controls (44).

The group of Kubes has proposed an alternative model for the non-lytic extrusion of genetic material from neutrophils, named “vital” NETosis, which occurs within 5 to 60 minutes of neutrophil stimulation (46–48). This process is independent of NADPH-derived ROS (47, 49) and is promoted by several stimuli: LPS-activated platelets (46); gram-positive bacteria such as *Staphylococcus aureus*, through TLR-2 and complement receptor 3 (C3R) stimulation (48); fungi such as *Candida Albicans*, through C3R activation (49). In vital NETosis, nuclei undergo rounding and decondensation, followed by separation of the inner and outer nuclear membranes to form a blebs-like structure and the budding of vesicles containing nuclear DNA, which are released in the extracellular space through exocytosis to originate NETs (47). According to this model, neutrophils survive as anucleated cytoplasts while still retaining their ability to crawl or exert phagocyte functions (48). Mitochondrial DNA is a negligible component of these early NETs induced by *S. Aureus* (47), but mitochondrial complex III and mtROS contribute to their formation (50).

In conclusion, these data indicate that the vital release of genetic material from neutrophils is always dependent on ROS originating from either NADPH (42) or mitochondria (44, 50). These extracellular DNA fibers have been proposed to boost autoimmune responses in SLE (43) or to exert anti-microbial functions (48). This process occurs *via* two different exocytosis mechanisms: one mainly involving oxidized mtDNA, and another involving nuclear DNA. According to this last hypothesis, neutrophils survive as anucleated cytoplasts, a condition not dissimilar from mature platelets, which are also devoid of nuclei and remain alive approximately for seven days in circulation (51). Interestingly, a recent report has shown that even necroptotic neutrophils can release NETs and occasionally persist as anucleate cytoplasts (34). However, further studies are necessary to confirm the possibility that neutrophils might survive as functionally active cells *in vivo* without the nucleus.

### DNA Extrusions From Other Innate Immune Cells

#### Eosinophils

The extrusion of ETs has been documented also in eosinophils - a granulocyte subset much less abundant than neutrophils - which perform effector functions in T helper (Th)2-mediated inflammation like in respiratory allergies (52), anti-parasite responses (52), but also CNS autoimmunity such as neuromyelitis optica (NMO) (53, 54). Similar to NETosis, two prevailing schools of thought have emerged in the field, one considering ETs formation as the result of a cell death program (55), and another proposing ETs release as an inflammatory process deployed by viable cells (56). Yousefi and coworkers have first shown that eosinophils primed with IL-5 or IFN-γ and exposed to LPS, C5a, or eotaxin extrude DNA fibers associated with granule proteins, such as eosinophil cationic protein (ECP) or major basic protein (MBP), extremely rapidly (in less than one second). Time-lapse confocal imaging analysis in this work revealed perinuclear, but not nuclear, structures as the source of the released DNA, which the authors identified as being of mitochondrial origin, based on *in situ* detection of the mitochondrial ATP synthase subunit 6 (*Atp6*) gene but not of nuclear glyceraldehyde-3-phosphate dehydrogenase (*Gapdh*) gene (56). These eosinophils-derived ETs entrap and kill *E. Coli in vitro* and originate in a ROS-dependent but cell death-independent fashion (56). Some years later, another group has found that also suicidal ETosis can occur in eosinophils upon activation for longer times (2 hours) with several stimuli such as immobilized IgG, IgA, the calcium ionophore A23187, PMA, GM-CSF or IL-5 plus platelet-activating factor (PAF), and *S. aureus* (55, 57). Like suicidal NETosis, eosinophilic ETosis is a lytic cell death program involving NADPH-oxidase, histone citrullination by PADs enzymes and leads to the secretion of histone-decorated DNA fibers that can ensnare pathogens such as *S. aureus* and *C. albicans* (55, 57, 58). Interestingly, lytic ETosis in eosinophils is associated with extracellular deposition of Charcot-Leyden crystals, protein structures composed of galectin-10 and generally present in tissues of eosinophil-related disorders, such as allergic and parasitic diseases and myeloid leukemia (59). ETs originated from eosinophils, decorated with histones and intact secretory granules are present in the secretions of patients with chronic eosinophilic rhinosinusitis or obstructive lung disease and in the tissues of subjects with eosinophilic granulomatosis with polyangiitis (57, 58, 60, 61). To fuel the controversy between the lytic and vital release of ETs, Gevaert et al. proposed that, in this same type of patients, the release of MBP+ ETs might occur in a NADPH oxidase-dependent manner, but in the absence of cellular breakdown (62, 63). The extrusion of histone-positive ETs associated with cell death by eosinophils occurs also in response to fungi such as *A. fumigatus*, however, these chromatin fibers do not exert antimicrobial or fungistatic activities (64). Interestingly, eosinophilic ETs induced in response to *A. fumigatus* originate independently of PAD4 but involve the Src family, Akt, calcium and p38 MAPK signaling pathways (65). More recently, the engagement of Dectin-1 receptor on eosinophils was found to trigger the extrusion of ETs composed of both nuclear and mitochondrial DNA fibers, ECP, acetylated histones, and citrullinated histones (66).

#### Basophils

Vital ETs release has also been proposed for basophils, the rarest granulocyte population that accounts for less than 1% of peripheral blood leukocytes and is mainly involved in Th2-orchestrated immune responses (67). Basophil-derived ETs are composed of mitochondrial but not nuclear DNA associated with granular proteins such as basogranulin and mouse mast cell protease 8 (68). These ETs are released independently by NADPH oxidase after priming with IL-3 followed by stimulation with C5a or IgE (68). Basophil-derived ETs have been proposed to counteract the growth of bacteria such as *E. Coli* and *S. Aureus in vitro* (68).

#### Mast Cells

Mast cells (MCs) are master players in allergic inflammation and immune defense against certain parasites or bacteria (69), but have also been involved in autoimmunity (70) and cancer (71). Mast cells release ETs of DNA decorated with histones, cytoplasmic or granular proteins such as tryptase (72), the anti-microbial peptide LL-37 (72) or β-hexosaminidase (73), in response to several stimuli including PMA (72, 73), hydrogen peroxide (72), monosodium urate crystals (74) or exposure to pathogens such as *Streptococcus pyogenes* (72), *Listeria Monocytogenes* (73), *Leishmania* (75), *Mycobacterium tuberculosis* (76). This process is dependent on NADPH oxidase (72, 76) and has been linked to cell death (72). MC-derived ETs have been suggested to counteract bacterial growth, however, the significance of these studies is restrained by the same limitations previously described for suicidal NETosis in the dedicated paragraph of this review. In the context of autoimmune disorders, MCs from human skin explants exposed to IL-23 and IL-1β or derived from psoriasis patients have been shown to release ETs decorated with chymase and IL-17, which might contribute to psoriasis (77). Interestingly, a vital release of nucleic acids has been documented also in the human MC line LAD2, which ejects mtDNA both free and stored in exosomes upon degranulation, in the absence of cell death (78). Furthermore, LAD2 cells treated with mitochondria isolated from the same cell line undergo degranulation and secretion of pro-inflammatory mediators, suggesting an autocrine effect promoted by the released mitochondrial components (78). It was not clarified in this paper whether part of the observed effects depended on mtDNA.

#### Monocytes/Macrophages

Preliminary evidence for ETs extrusion from monocytes/macrophages has been obtained with human and murine macrophage cell lines exposed to statins (79). More recently, it was demonstrated that human monocytes purified from peripheral blood release ETs in a NADPH oxidase-dependent fashion when stimulated with classical NETotic stimuli such as PMA, the calcium ionophore A23187, PAF, and the fungal component zymosan (80). Monocytic ETs are constituted of both mitochondrial and nuclear DNA and are endowed with procoagulant properties *in vitro* (80). It should be noted that proteins classically associated with NETs, such as MPO, elastase, and CitH3, have been also identified in ETs derived from monocytes (80), thus suggesting that caution should be paid to assuming the involvement of NETs in a given pathological context, simply relying on immunostaining for CitH3, elastase, and MPO in the target tissue. Macrophages can extrude ETs in response to several microorganisms, towards which ETs exert anti-microbial effects (10). Mitochondrial and nuclear DNA, histone H4, elastase and MPO constitute ETs derived from the macrophage line THP-1 stimulated with *Mycobacterium Massiliense*. Interestingly, these ETs, which do not require NADPH oxidase but depend on calcium influx, favor bacterial growth and aggregation (81).

#### Microglial Cells

Recent work has pointed out that even microglial cells, CNS resident phagocytes, release ETs upon exposure to several stimuli including PMA, ionomycin, *Listeria monocytogenes* and dopamine (82, 83). Microglial ETs are composed of DNA decorated with histones, matrix metalloproteinases (MMP)-9 and -12, PAD2, or MPO and have bactericidal properties (82, 83). PMA or *L.monocytogenes-*induced ETs rely on NADPH-oxidase and PADs activity and originate from microglial vesicles (82), while dopamine-triggered ETs form independently of ROS and apparently also of cell death (83). However, further studies are necessary to clarify whether microglia undergo suicidal or vital ETosis.

### Extracellular DNA Threads From Lymphocytes

Even though the release of ETs has been originally identified in innate immune cells, it’s not uncommon that innate immune mechanisms can intrinsically affect the activity of adaptive immune cells, as is the case for TLRs (84) or NLRP3 inflammasome (85), which importantly modulate T cell effector functions.

Traces of extracellular DNA have been detected first in T and B cell cultures exposed to stimuli such as ionomycin or anti-IgM plus LPS (86). However, as discussed by the same authors, it was not clear whether these strands derived from a process similar to ETosis or any other form of cell death, as the protein composition of these DNA extrusions was not assessed in this work (86). We have recently shown that highly purified mouse naïve CD4+ T cells stimulated with α-CD3/α-CD28 Abs extrude DNA fibers composed of both narrow and, less frequently, thicker nucleic acid strands (87). These DNA threads were dotted with CD4 marker and stained with two mitochondrial probes, namely MitoTracker and MitoSOX Red, potentially suggesting the presence of oxidized mtDNA in these structures, while histones were only occasionally detected (87). The DNA strands derived from mouse naïve CD4+ T cells support their effector functions in an autocrine way and their formation is importantly reduced in the presence of a mtROS scavenger which, in parallel, also lessens the production of pro-inflammatory cytokines (87). As T helper cells are more orchestrators of anti-microbial responses rather than direct executioners of pathogen killing, these DNA extrusions were studied not in relation to an anti-microbial function, i.e. pathogen entrapment, but to the intrinsic activity of T cells and were named T-helper-released extracellular DNAs (ThREDs) (87). Whether this phenomenon occurs through vital or lytic mechanisms has not been directly assessed in this work. However, while the incubation of activated naïve mouse CD4+ T cells with a mtROS scavenger hampered ThREDs formation, the percentage of dying cells was unaffected by this treatment, suggesting the possible occurrence of a vital mechanism of extrusion (87). Subsequent work has shown that a subset of human Th17 cells reactive to *Cutibacterium acnes* and stimulated with α-CD3/α-CD28 Abs or PMA in the presence or absence of *C. acnes*, release ETs composed of DNA and histone H2B which ensnare bacteria *in vitro* (88). Interestingly, Th17 cells not specific for *C. acnes* or Th1 and Th2 subsets were unable to form ETs, suggesting a specific activation state of Th17 cells responding to bacteria (88). Another report has found that human CD4+ T cells stimulated with α-CD3/α-CD28 Abs form a halo rather than filaments of DNA around cells (89). In contrast, a portion of human CD8+ T cells activated with α-CD3/α-CD28 Abs was found to extrude long DNA fibers that connect cells with intact morphology and are positive for the vesicle marker CD107a (89). The nuclear or mitochondrial origin of these DNA extrusions was not investigated in this work.

B cells rapidly eject mtDNA webs when stimulated with synthetic CpG and non-CpG oligonucleotides of class C, but not when treated with classical NETotic stimuli such as PMA or LPS (90). Interestingly, low amounts of mtDNA have been detected also in supernatants of unstimulated B cells, similar to resting neutrophils (43). The release of mtDNA from B cells is not impaired by inhibitors of apoptosis, necrosis, necroptosis, or autophagy and is not dependent on ROS (90). These extracellular mtDNA fibers are not associated with TFAM or antimicrobial proteins and, when incubated with *E. Coli*, do not exert any anti-bacterial effect, but they are highly interferogenic for PBMC. DNase-mediated disruption of these DNA webs increases the production of IFN-α by PBMC and it has been hypothesized this is due to the higher interferogenic potential of smaller DNA fragments (90). As CpG-C sequences are synthetic stimuli, other work is necessary to understand whether and how this process might occur under physiological circumstances.

**Figure 1:**
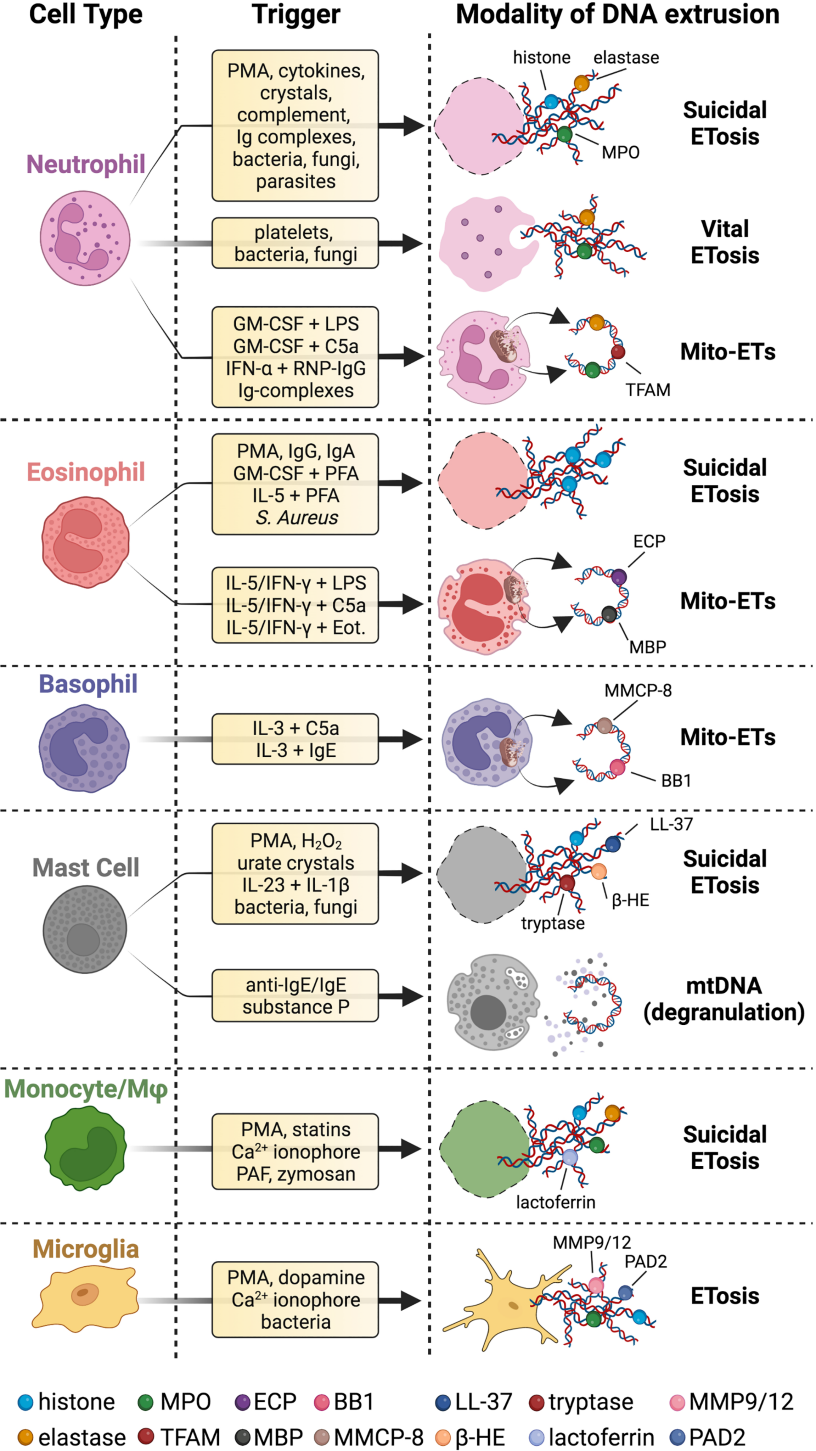
Innate immune cells extrude different types of DNA strands and with several modalities. Upon stimulation with a wide range of stimuli, innate immune cells release chromatin fibers coated with proteins in a cell death program called suicidal ETosis. Neutrophils can also proceed to ETs extrusion surviving as anucleated cytoplasts that can still exert immune effector functions (vital ETosis). Innate immune cells can even secrete ETs of mtDNA coated with cytoplasmic or granular proteins (Mito-ETs). Mast cells have been reported to release mtDNA concomitantly with degranulation. Microglial cells release ETs, but it is not clear whether this process is associated with cell death. Specific references to different ETs stimuli and types are given in the body of the text. BB1 = basogranulin; β-HE = β-hexosaminidase; Eot. = eotaxin; MMCP-8 = mouse mast cell protease 8. Created with BioRender.com.

**Figure 2:**
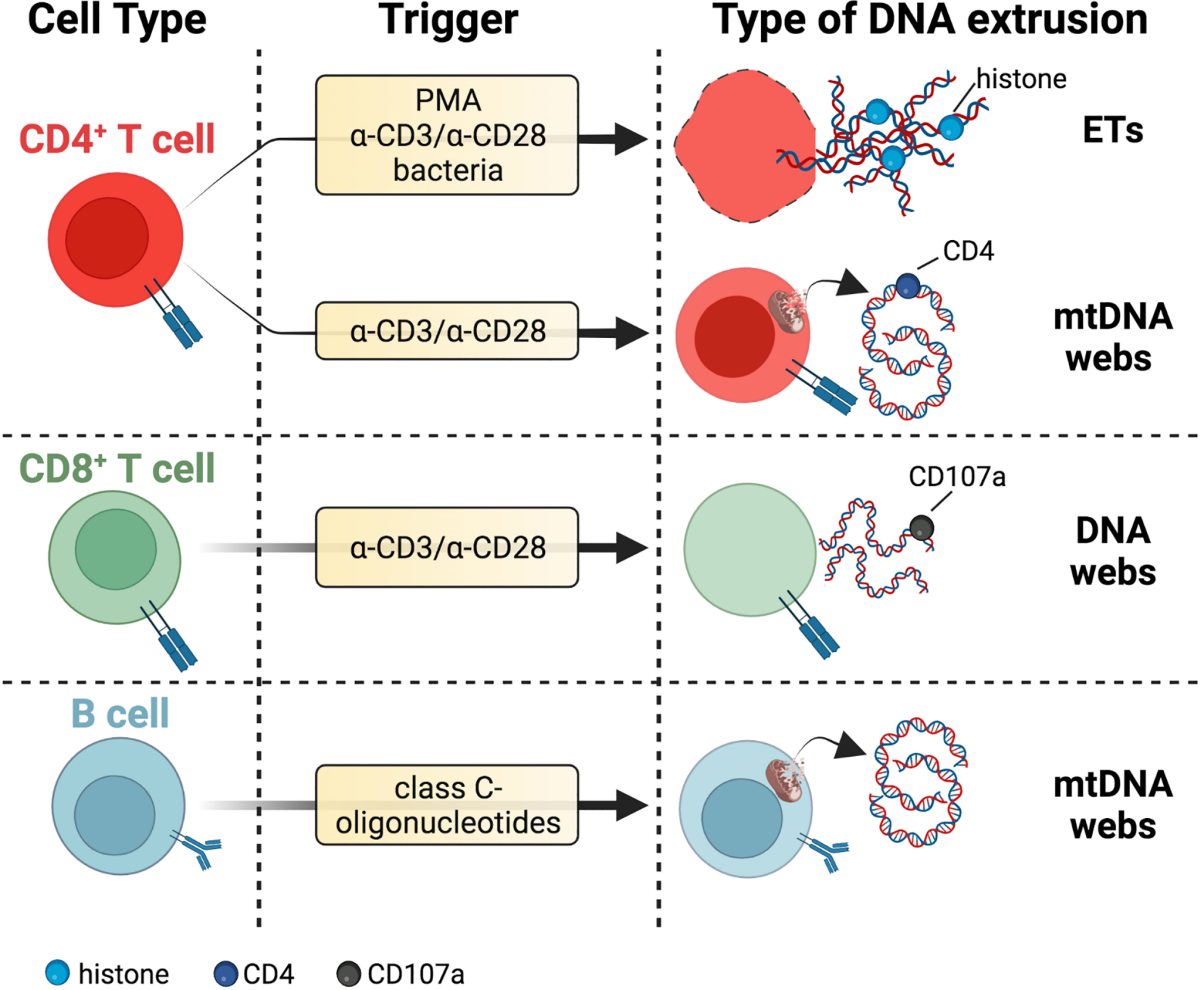
DNA threads released by adaptive immune cells. T cells secrete DNA after activation with polyclonal stimuli such as α-CD3/α-CD28 antibodies or PMA. These extrusions include thick DNA strands coated with histones reminiscent of ETs or narrow DNA threads dotted with CD4 marker and potentially of mitochondrial origin due to staining with mitochondrial probes MitoTracker and MitoSOX Red. DNA fibers released by activated CD8+ T cells colocalize with the vesicular marker CD107a. The lytic or vital nature of DNA release from T cells needs further investigation. B cells eject interferogenic mtDNA webs after incubation with class C-oligonucleotides, but these fibers are not associated with TFAM or antimicrobial proteins. Specific references are given in the body of the text. Created with BioRender.com.

A schematic representation of the different stimulating factors and types of extracellular DNAs released by immune cells is depicted in **Figure 1** for innate immunity and **Figure 2** for adaptive immunity.

## Extracellular Traps in Neurodegenerative Diseases

### Alzheimer’s Disease

Alzheimer’s disease (AD) is a progressive degenerative brain disorder and the most common form of dementia, affecting more than 35 million people worldwide (91). The neuropathological hallmarks of the AD brain consist of abundant β-amyloid senile plaques, neurofibrillary tangles, dystrophic neurites with hyperphosphorylated tau, cerebral amyloid angiopathy, synaptic dysfunction, and neuronal loss. This pathological scenario is accompanied by strong neuroinflammatory responses involving CNS-resident and peripherally-derived immune cells, the release of immune mediators, vascular inflammation, and blood-brain barrier (BBB) dysfunction (92–94). Overall, it is now widely accepted that neuroinflammation in AD actively contributes to the pathogenesis of the disease (92–94). BBB leakage and the presence of blood-derived leukocytes, including lymphocytes, monocytes, and neutrophils, have been extensively reported in the brain of AD patients and in corresponding experimental models (95). Although most studies suggest that Aβ and tau contribute to blood vessel abnormalities and BBB damage (95), more recently it has been proposed that BBB rupture precedes the deposition of parenchymal senile plaques and the appearance of perivascular tau in the hippocampus of both human AD and animal models (96). This hypothesis suggests, on the one hand, that BBB breakdown could be an early biomarker of human cognitive dysfunction independent of Aβ and tau, and that infiltration of immune cells from the periphery might play a key role in AD inflammation and neurodegeneration, on the other. Since innate myeloid cells are invariably found in close proximity to Aβ plaques within the AD brain (97), much attention has been primarily devoted to the possible role of monocytes and neutrophils in AD. Circulating monocytes can infiltrate the brain in a C-C chemokine receptor type 2 (CCR2)-dependent manner, differentiate into microglial-like cells, and clear vascular Aβ in APP/PS1 mice (98). However, this influx of peripherally-derived myeloid cells fails to modify the long-term cerebral β-amyloidosis and brain function in APP23 and APP/PS1 mice models (98). As far as we know, no reports on the possible release of ETs from monocytes have been described in AD.

More recently, Zenaro and colleagues have shown that neutrophil brain accumulation and the production of endovascular and intra-parenchymal NETs in areas adjacent to Aβ deposits might cause chronic dysfunction and inflammation of the BBB, thus contributing to the progression of AD-like pathology in two different AD models, the 5xFAD and 3xTg-AD mice (99). Briefly, the Aβ deposits themselves would enhance the lymphocyte-function-associated antigen-1 (LFA-1)-dependent neutrophil adhesion and extravasation by triggering the transition of LFA-1 integrin from low- to a high-affinity state. The administration of LFA-1- or neutrophil-targeting Abs significantly ameliorated both cognitive decline and brain inflammation in these models by affecting microglial activation and IL-17 secretion. Comparative analysis on AD brain revealed that neutrophils adhere to and spread inside the cortical brain vessels and release NETs in vasculature and parenchyma. The rebounds of this striking study are the followings. First, it suggested a key role of Aβ plaque in the recruitment of neutrophils and then outlined novel inflammatory paths underlying the pathophysiology of AD. Indeed, the formation of intravascular and parenchymal NETs could exacerbate BBB dysfunction, which, in turn, could facilitate the infiltration of peripheral immune cells. More recently, the ratio between C-X-C chemokine receptor type 4 (CXCR4)+ harmful and hyperactive neutrophils to CD62L+ senescent neutrophils and the levels of ROS in blood were found significantly higher in AD patients with severe dementia when compared to mild cognitive impairment (MCI) patients (100). These data suggest that the neutrophil phenotype could reflect the rate of cognitive decline in AD patients and may represent a possible non-invasive biomarker of disease progression in AD patients. Third, the significant reduction of microglial activation exerted by LFA-1 blockade highlights a potential neutrophil-microglia vicious cycle where neutrophil-derived IL-17 might directly damage neurons and promote the release of inflammatory mediators by microglia, which in turn might trigger further NETs release in the AD brain (100). Moreover, amyloid-β activates NADPH oxidase (101), a key enzyme mediating the extrusion of NETs, whose constituents cleave extracellular matrix proteins (102) and activate the NLRP3 inflammasome pathway, eventually leading to cell death (103). Finally, intravascular NETs can promote thrombosis, which further exacerbates brain microvessel pathology, and cause direct toxic effects on the endothelium due to the release of proteolytic proteins, such as NE, MMPs, and cathepsin G.

A new perspective regarding a mutual relation between NETs and the complement system in AD has been recently highlighted (104). A role for the complement pathway in AD neuropathology was hypothesized more than three decades ago, and the results of a significant number of studies are consistent with the involvement of this pathway in AD pathogenesis (104). C1q and several other complement factors are associated with fibrillar Aβ plaques, and their synthesis has been described within the AD brain (105). According to this novel view, NETs and neutrophils may activate the alternative complement pathway through C5a factor by its cognate C5aR1 receptor, leading to further neutrophil recruitment to the Aβ plaques and additional release of NETs (104). In parallel, C1q would bind to Aβ plaques and activate the classical complement pathway, favoring a pathological circuitry between inflammatory and neurodegenerative processes. Recently, studies in bacteria showed that complement opsonization promotes NETs release through CR1 on neutrophil membranes (106). Whether a similar process happens in AD patients has not been established yet, however, this possibility is not far-fetched. Indeed, increased susceptibility to late-onset AD has been associated with CR1 polymorphisms (107) and high serum levels of soluble CR1 have been recently reported in AD patients (107).

Even though the formation of NETs might provide an explanation for several aspects of neuroinflammation in the AD brain, the precise mechanisms underlying NETosis and how NETs components might perpetuate inflammatory processes in AD are far from being clear and need further studies. Whether NETs could promote the release of neoantigens triggering autoantibody production is conceivable although not verified yet (for an extensive review see (108)). It’s also plausible that other components of the senile plaques, in association or not with Aβ, could actively promote neutrophil recruitment and NETs release. Due to their high affinity for Aβ as either soluble or fibrillary peptides, cholesterol and apolipoprotein e (apoE) are important constituents of the senile plaques (109, 110). Cholesterol crystals are known to act as danger signals in the atherosclerotic plaques through NLRP3 activation and IL-1β secretion and have been recently shown to trigger NETs release (31). Whether and how cholesterol could prime NETs formation also in cerebral amyloid deposition diseases is an intriguing hypothesis to be verified.

Furthermore, the accumulation of amyloid fibrils by specific misfolded proteins is not limited to AD, but it is a shared feature of several types of amyloidosis in humans, including chronic neurodegenerative disorders such as Parkinson’s disease (PD) and prion-associated diseases. A recent *in vitro* study revealed that amyloid fibrils obtained from α-synuclein, an amyloidogenic protein implicated in the formation of radiating filaments within Lewy bodies in PD, induce NADPH-oxidase dependent NETs formation (111). Moreover, the authors showed that NETs-associated elastase digest amyloid fibrils into short cytotoxic fragments, suggesting that amyloid fibrils themselves might act as a reservoir of toxic oligomers exacerbating amyloidosis. To our knowledge, no data on ETs in PD or prion diseases have been reported so far. In light of the involvement of NETs in amyloidosis, whether they could play a pathogenic role also in PD and/or prion disease would deserve further investigation.

### Amyotrophic Lateral Sclerosis

Amyotrophic lateral sclerosis (ALS) is a progressive and fatal neurodegenerative disease that affects both upper and lower motor neurons (MNs) and leads to muscle weakness and ineluctable progression of paralysis after disease onset. Currently, there are no effective therapies for ALS and the survival after diagnosis ranges from 3 to 5 years (112). Experimental and clinical evidence suggests that lower MNs pathology in ALS begins at the distal axon and proceeds in a so-called “dying back” pattern (113, 114). Impairment of MN function significantly precedes the onset of clinical signs (shaking, tremor) and the beginning of motor neuron loss. Indeed, at early disease stages, the neuromuscular deficits do not result from motoneuronal cell death but rather from loss of axonal integrity. Neuroinflammation involving infiltration of lymphocytes and macrophages, glial activation, and complement deposition has been reported in ALS (112). However, the inflammatory processes that contribute to peripheral MN deficit are still elusive compared to those involving the CNS. Moreover, although it is generally accepted that monocytes infiltrate the peripheral nerves and skeletal muscles of both ALS patients and transgenic mouse models (115), whether they could be deleterious or protective is a matter of controversy (116, 117). Similarly, the impact of other immune cells, such as mast cells and neutrophils, on MN degeneration is still unclear. A recent outstanding study by Trias and colleagues (118) has found that quadriceps muscles from ALS subjects are characterized by massive infiltration of degranulating MCs interacting with neutrophils positive for NE and forming large ETs. The infiltration of MCs, phagocytic neutrophils, and NETs formation are also abundant in the neuromuscular junction, in the proximity of degenerating axons and motor endplates, and is markedly associated with paralysis progression in the symptomatic SOD1G93A rat model. The administration of masitinib (119), a selective blocker of the c-kit-mediated pathway that inhibits MC function, potently prevents immune cell infiltration, deposition of NE and NETs formation, ameliorating axonal pathology, secondary demyelination, and the loss of myofibers. As far as we know, this study has provided the first evidence that MCs and NETs may directly promote tissue damage and degradation of muscular components in ALS, offering a novel pathogenetic perspective with significant implications for setting up innovative therapeutic strategies. Moreover, since also MCs release ETs (72), the contribution of MC-derived extracellular DNAs to ALS pathology represents an interesting aspect to deepen in future studies.

## Extracellular Traps in the Injured CNS

### Ischemic Stroke

Ischemic stroke (IS) is a hypoxic-ischemic disorder resulting from a clot obstruction of the cerebral flow and leading to the formation of an ischemic core and surrounding ischemic penumbra. Ischemic stroke is one of the primary causes of death and long-term disability with limited therapeutic options (120). From a pathogenic perspective, IS can be conceived as a multiphasic process where the initial ischemic insult evolves over several hours or days into the penumbral areas, expanding the injury to the entire ischemic region (120). To date, the mainstay of IS treatment is the rapid pharmacological thrombolysis with tissue plasminogen activator (t-PA) or endovascular clot removal (thrombectomy) to allow for early reperfusion, limit tissue damage and improve the clinical outcome (121). The double-edged role of the immune system in stroke pathophysiology has become increasingly evident and it is now established that cerebral ischemia encompasses an acute inflammatory stage sustained by the innate immunity, and a chronic phase involving adaptive immune cells. For a recent comprehensive review [see ref (122)].

Key hallmarks of the acute immune response in IS are the damage of the BBB, the immediate secretion of brain-generated DAMPs and cytokines, and the recruitment of leukocytes to the brain. The initial transmigration of immune cells occurs in a peculiar temporal and spatial pattern through different routes such as the activated parenchymal and leptomeningeal vessels and the choroid plexus (123–126). Noteworthy, recent work revealed the migration of myeloid cells through microscopic vascular channels that traverse the inner skull cortex in the murine IS model, implying that - besides the periphery - the skull bone marrow, through the meninges, represents a relevant source of immune cells in IS (127). Several studies on experimental models have shown that neutrophils and monocytes are quickly engaged at injury site by increased local secretion of GM-CSF (127). In particular, two-critical steps have been recognized in neutrophil activity during ischemia: the intravascular and intraparenchymal phases. In the early and often protracted intravascular inflammation, TLR-4 engagement on neutrophils triggers degranulation with the release of proteases including NE and MMP-9, ROS, reactive nitrogen species, perforin, cytokines, and NETs. The intraparenchymal phase requires IL-1-dependent neutrophil endothelial transmigration by Mac1 (CD11b/CD18) and ICAM-1 adhesion, which triggers the acquisition of a cytotoxic phenotype (named N1 phenotype) with the secretion of proteases associated with decondensed DNA (128). Inhibiting neutrophil migration with anti-Mac1 antibody promotes a significant reduction in the infarct volume in rat middle cerebral artery occlusion (MCAO) reperfusion model. However, the Acute Stroke Therapy by Inhibition of Neutrophil (ASTIN) clinical trial has reported no clinical improvement in acute ischemic stroke patients (129), thus suggesting a prominent role for intravascular neutrophil toxicity in the acute IS. A previous study indicated that IL-1-driven cerebrovascular transmigration promotes a neurotoxic phenotype in neutrophils leading to rapid neuronal death comparable to that obtained by treating neurons with NMDA (128). Transmigrated neutrophils had an increased pro-inflammatory profile, with a reduction in membrane-associated CD62L (L-selectin), indicative of L-selectin shedding and cell activation, as well as significantly increased expression of neutrophil C-X-C motif chemokine ligand (CXCL)1 and IL-6. Nevertheless, DNase treatment of conditioned medium from transmigrated neutrophils did not result in any improvement of neuronal loss in cell cultures, thus indicating that neurodegeneration could not be attributed merely to the presence of neutrophil-derived extracellular DNAs but rather required neutrophil degranulation (128). Of note, Cuartero et al. recently showed that nuclear peroxisome proliferator-activated receptor (PPAR)-γ receptor activation during brain inflammation can switch neutrophils towards a neuroprotective N2 phenotype (Arginase-1+ and CD206+) associated with enhanced clearance of tissue debris that could be beneficial in IS (130). In line with these findings, PPAR-γ activation by Rosiglitazone promotes significant protection and improved functional outcome in the MCAO model (131).

In this model, the route of transmigration of CitH3+ neutrophils after injury and the progression of NETosis starts from the leptomeningeal vessels, passing through Virchow-Robin spaces to eventually reach the brain parenchyma one day after the injury, where they exacerbate neuronal damage by releasing HMBG1 (132). The latter triggers the production of further CitH3 *via* CXCR4 and TLR-4 and contributes to the formation of additional NETs, establishing a vicious cycle between neuronal death and NETosis. Consequently, NETosis suppression in the ischemic brain by intranasal administration of a PAD4 inhibitor delays inflammation and improves the repair process, as confirmed by reduced immune cell infiltration and enhanced blood vessel formation (132).

Remarkable attention has been paid to the possible pathogenic role of NETs in IS since Laridan and coworkers first described the presence of abundant dense NETs containing CitH3 originating from CD66b+ granulocytes in thrombi extracted from the cerebral circulation of IS patients (133). The presence of NETs has been later confirmed in ipsilesional brain tissue from ischemic stroke patients (134). Of note, *ex vivo* lysis of fresh thrombi collected from patients with a combination of t-PA and DNase is significantly more effective than t-PA alone, providing a proof of concept that targeting NETs could be a novel pro-thrombolytic strategy. The histone-DNA complexes might wrap fibrin and increase its mechanical stability and stiffness in clots, compromising t-PA-induced thrombolysis in IS, as already described in thrombosis (135). Structural analysis of stroke thrombi revealed that NETs contain tissue factors such as Willebrand Factor and histones, serving as a scaffold for platelet adhesion, activation, and aggregation (136, 137). The characterization of the internal carotid atheromatous plaques in patients revealed that NETs, by binding platelet-derived microparticles, might promote the assembly of thrombi and trigger a pro-coagulant activity of the endothelial cells through the exposition of coagulation factors, such as phosphatidylserine and tissue factor (138). It could be hypothesized that the intravascular NETs formation might promote the resistance to fibrinolytic therapy, reported in more than half of IS patients treated with t-PA (121), and might contribute both to the no-reflow phenomenon and secondary micro-thrombosis (121). Moreover, a recent work has shown that antibody-mediated depletion of platelets or HMGB1 genetic deletion in platelets impairs NETs formation and lessens ischemic stroke brain injury in transient MCAO mouse model (134). The administration of neonatal NET-inhibitory factor (nNIF), a novel NET inhibitor peptide, significantly ameliorates brain damage and motor function in this model when injected within 1h after stroke onset (134). Previous studies have demonstrated that *in vitro* treatment with DNase cleaves NETs-platelet interaction (139). DNase administration in a transient MCAO mouse model exerts a protective effect against ischemic injury, resulting in improved neurological and motor tests (140). Furthermore, NETs disruption with DNase elicits clot burden reduction and hinders thrombin generation and activity (141). Noteworthy, a recent work pointed out that t-PA treatment in mice with thrombotic stroke increases NETs, which amplify BBB damage, thus enhancing cerebral hemorrhage compared to the control group. This detrimental effect is counteracted by DNase treatment or *Padi4* deficiency. Putative t-PA adverse effects were recently attributed to the induction of the DNA sensor cGAS-STING pathway in microglial cells and consequent increase of downstream signals pTBK1, pIRF3, and IFN-β in the ischemic cortex after stroke (142). Both DNase I treatment and deficiency of *Padi4* or *cGas* in mice reversed tPA-associated brain hemorrhage after ischemic stroke by affecting the production of IFN-β. Overall, recent literature has provided a bulk of evidence regarding the pathogenic role of neutrophils and NETs in acute stroke, highlighting their contribution to both obstruction of cerebral vessels and neuronal damage. However, whether they could contribute to the cerebral and systemic immune changes in the chronic phase of stroke needs to be investigated.

The presence of neutrophils (MPO+ cells) and NETs (CitH3+) was recently evaluated in cerebral thrombi of different stroke etiology, i.e., cardioembolic, large artery atherosclerosis, or undetermined cause, retrieved by endovascular thrombectomy (143). Although no differences are observed in the number of neutrophils among clots of diverse origin, NETs are significantly more abundant in cerebral thrombi of cardioembolic origin compared to those resulting from large artery atherosclerosis. Of note, plasma NETs levels positively correlate with NETs content in thrombi at the stroke onset. These data confirm the presence of NETs as an essential component of thrombi, as widely described above, and provide additional insight for discriminating ischemic strokes of different etiology. Further, the positive correlation between circulating NETs and their content in the thrombus might open a new perspective in search of a plasmatic thrombus biomarker.

The release of extracellular DNA is not an exclusive prerogative of neutrophils (12). In addition to neutrophils, cells like monocytes, mast cells, and even eosinophils can release extracellular DNA, as described above. In this regard, a recent study outlined the role of eosinophil ETs in atherosclerosis and thrombosis. Marx and colleagues demonstrated that eosinophils foster platelet activation and can interact directly with platelets after endothelial injury and during thrombus formation in a mouse model of arterial thrombosis (144). Thrombus analysis identified the presence of MBP-coated extracellular DNA, clearly indicating the presence of eosinophilic ETs, which sustain thrombus formation by promoting platelet activation. Interestingly, this process was dismantled using an anti-P-selectin antibody. In addition, pretreatment with Siglec-F, a potent eosinophil inhibitor, significantly decreased thrombus stability in an experimental model of arterial thrombosis, suggesting that targeting eosinophils could also represent a promising approach in the prevention and therapy of atherosclerosis and thrombosis (144). Whether eosinophilic ETs may also play a pathogenic role in acute IS in either patients or experimental models has not been reported yet.

### Traumatic Brain Injury

Traumatic brain injury (TBI) is a leading cause of morbidity and disability worldwide. Only in the US, about 2 million people reported TBI events, contributing to 30% of all injury-related deaths (145). Tissue damage heterogeneity and the diversity of injury-related mechanisms make the treatment of this disorder particularly challenging. The pathogenesis of TBI involves two components. The initial mechanical injury is characterized by multiple pathological alterations such as intracranial hemorrhage, epidural and subdural hematoma, axonal and dendrite injury, glial activation, microvasculopathy with microbleeds, endothelial damage, and BBB breach (146). In minutes to months and far beyond the starting insult, a secondary injury occurs and involves several pathways including excitotoxicity (147), lipid peroxidation due to steady ROS generation (146), and a complex neuroinflammatory response involving both innate and adaptive immunity (148). In this complex scenario, neuroinflammation is widely accepted as an important and tunable component of the secondary injury response. However, it is essential to understand the timing of the immune response and the specific involvement of the intra-parenchymal or vascular compartments in TBI, as inflammation evolves over time, exerting both beneficial and detrimental effects. Similar observations can be also made for spinal cord injury (SCI), a topic addressed below. Increased intracranial pressure (ICP) due to cerebral edema after mechanical trauma is well documented in mild to severe TBI forms in both humans and animal models and is a recognized cause of poor clinical prognosis. Increased HMGB1 has been associated with elevated ICP in patients and promotes cerebral edema after TBI in mice *via* TLR4-activation (149). Consistent with this, prompt TLR-4 inhibition (0.5-4 hours after TBI induction) with VGX-1027 attenuates both microglial-driven neuroinflammation and neurovascular dysfunction in mice (149).

Several studies have shown that neutrophils can roll along inflamed cerebral vessels within 5 minutes after head trauma and enter the subarachnoid and subdural spaces within 4 hours after injury, thus promoting cerebral hypoperfusion and contributing to both microcirculatory/BBB disruption and edema formation (149). In 2010, Rhind et al. showed a significant surge in peripheral neutrophil numbers in the early hours after TBI, lasting until 48 hours post-injury (150). More recently, Vaibhav and colleagues reported the presence of TLR-4 and PAD4-dependent NETs and cerebrovascular dysfunction in a controlled cortical impact (CCI) TBI mouse model (151). Through transmission electron microscopy analysis and immunogold labeling, this work revealed the presence of CitH3+ and NE+ NET-like structures adjacent to injured blood vessels in the pericontusional brain cortex at 24 hours after trauma. Infiltrating neutrophils exhibited both high TLR-4 and PAD4 expression and increased extracellular MPO and NE deposition, all features affected in mice lacking functional TLR-4 and mice treated with a PADs inhibitor or DNase. In particular, DNase administration to injured animals alleviated secondary cerebral edema and improved perfusion, suggesting that NETs can mediate cerebral hypoperfusion and edema formation after TBI. In parallel, the evaluation of endogenous DNase-I function and circulating NETs levels was performed in sera from patients with severe neurotrauma requiring cerebrospinal fluid diversion due to elevated ICP. Interestingly, in sera from human TBI patients, DNase-I activity inversely correlated to both ICP score and circulating NETs levels, as measured by MPO-DNA array. Overall, the data from this study suggest that NETs can propagate the formation of non-canonical microthrombi, eventually increasing ICP and cerebral edema, and suggest that restoration of serum DNase-I could provide a possible therapeutic opportunity in patients with elevated ICP after TBI. This hypothesis is in line with a recent study reporting that the presence of NETs in intravascular clots of patients with severe bacterial infections is associated with a defective host DNase, which is unable to degrade NETs in *ex vivo* assays (152). Interestingly, significantly higher levels of cell-free DNA and reduced DNase activity have been reported more recently in plasma obtained from TBI patients with poor clinical outcome (153). Since extracellular DNA/histones complexes have been shown to support plasma thrombin generation by platelet activation and impair fibrinolysis in sepsis (154), it is possible that similar pro-thrombotic properties could also occur in the contused brain as described above for IS.

The presence of the antimicrobial LL37 peptide in CitH3+ NETs has recently been reported within the periventricular nuclei in a rat model of diffuse axonal injury (155). In microglia/neutrophil co-cultures, LL37 secretion by neutrophils promotes IL-1β release from microglial cells through LL37-P2X7R interaction and activation of serine/threonine kinases MST1 signaling pathways. The authors suggest that prompt IL-1β release increases and/or modulates neurotransmitter levels and functions and ultimately contributes to secondary paroxysmal sympathetic hyperactivity, a relatively common complication secondary to severe TBI. Although the possible cross-talk between NETs and LL37-induced sympathetic excitation in TBI is intriguing, to date, further studies are needed to corroborate this hypothesis. P2X7 receptors are purinergic cation channels sensitive to high concentrations of ATP in the extracellular space, and have essential roles in inflammation, such as promoting neutrophil infiltration in the CNS. However, P2X7R is expressed also by oligodendrocytes, astrocytes, and ependymal cells (156). Their activation triggers an inflammatory cascade that includes the expression of metalloproteinases ADAM10/ADAM17 and the secretion of a plethora of inflammatory mediators, such as prostaglandin E2, IL-1β, IL-2, IL-4, IL-6, and TNF-α (157), which could differentially contribute to both sympathetic discharge and immune responses after brain injury. Similarly, *in vitro* experiments revealed that, in addition to IL-1β, LL37 can also induce the release of IL-6 and IL-8 by astroglial and microglial cells (158), which exert opposite effects on neuronal excitability. Furthermore, the involvement of purinergic signaling in TBI appears to depend on both the timing of observation after trauma and the severity of the injury. Using intravital imaging in a mild focal brain injury model (1-3 hours after injury induction), Roth and coworkers demonstrated that neutrophils are recruited more exclusively into the meninges and perivascular spaces in a P2X7R- and formyl peptide-receptor-dependent manner, but not in the injured brain parenchyma (159). Transcranial administration of P2X7R antagonist 15 minutes after the injury results in enhanced meningeal cell death 12 hours later but has no impact on parenchymal damage compared to untreated injured mice. Thus, besides their inherent pathogenicity, early neutrophil recruitment to the damaged meninges after mild TBI could promote an immediate neuroprotective immune response (159). More recently, Oggioni and colleagues showed that long pentraxin PTX3, a pattern recognition molecule involved both in neurogenesis after secondary damage in TBI (160) and BBB integrity in stroke (161), is localized in the proximity of neutrophils and of NET-like structures only in the acute phase of CCI mouse model, suggesting that NETs could contribute via-PTX3 to the subacute stage of TBI (162).

The infiltration of neutrophils into the injured brain is primed by several stimuli including purines, chemoattractant molecules, and cytokines and is enhanced by the combined expression of vascular adhesion molecules. While it is generally accepted that polymorphonuclear cells are the first peripheral cells to reach the injured brain at vascular margination and meninges within hours after TBI, studies in contused patients have revealed that neutrophil invasion of the parenchyma occurs 3-5 days after trauma concomitantly with T lymphocytes (163). Depletion of granulocytes with anti-Gr1 Ab in CCI mice results in decreased brain edema and reduced numbers of active caspase-3+ cells (a readout of cell death), without affecting BBB dysfunction (164). However, the effect of anti-Gr1 Ab on granulocyte and myeloid subtypes was not verified in this work. Similar results were obtained with CXCR2-deficient mice (165). These data are apparently in conflict with previous ones by Whalen et al. (166), who instead reported that selective depletion of systemic neutrophils with anti-RP3 antibody in CCI rats does not affect either edema or BBB dysfunction after injury. Questions remain as to whether other mechanisms than those mediated by neutrophils and NETs may affect BBB permeability early after CCI.

### Spinal Cord Injury

Spinal cord injury (SCI) is a traumatic and detrimental condition resulting in temporary or permanent paralysis in affected patients, with a global incidence of about 20-25 cases per million worldwide (167). As in TBI, initial trauma in SCI triggers a series of molecular changes at the tissue and cellular level that lead to a secondary phase resulting in permanent damage and neurological dysfunction (168, 169). Due to scar formation, current treatments are far from satisfactory and targeting the secondary injury response remains critical to providing a clinical recovery. So far, administration of corticosteroids in the first few hours after injury remains the most effective approach to reduce the extent of injury and limit motor deterioration in patients (170).

Neutrophil recruitment to the injured SC occurs within hours after trauma, reaching a peak in 1-3 days after the injury, and much attention has been paid to their pathogenic role in SCI. The activation of neutrophil PRRs by locally-produced DAMPs (171) results in a dramatic upregulation of pro-inflammatory cytokines, proteases and tissue-degrading enzymes, overall contributing to a harmful tissue environment (172). More recently, one study highlighted the detrimental effect of NETs formation in a clip-compression SCI model (173). Infiltrating neutrophils at lesion sites in the spinal cord release NETs, as measured by dsDNA dye Sytox, CitH3, and MPO staining, exacerbating both secondary fibrotic scar formation and the disruption of blood-spinal cord barrier, promoted by an increased endothelial expression of the transient receptor potential vanilloid type 4 (TRPV4), a calcium-permeable nonselective cation channel. The inhibition or degradation of NETs by treating SCI-injured animals with DNase or PADs inhibitor provided a global improvement by limiting cell death and scar formation and promoting motor function recovery. In line with these data, pharmacological or genetic suppression of TRPV4 has been recently shown to prevent the degradation of tight junction proteins, preserve blood-spinal cord barrier integrity, and attenuate tissue scarring in SCI mice (174).

However, although neutrophils are generally considered detrimental in SCI, preventing their early vascular recruitment by anti-Gr1-depleting Ab surprisingly impairs functional recovery in a mouse model due to enhanced CXCL1 and chemokine (C-C motif) ligand (CCL)2/9 production (175). Similarly, Gasemlou et al. showed that the over-expression of secretory leukocyte protease inhibitor (primarily found in astrocytes and neutrophils) in transgenic mice with SCI or the administration of recombinant secretory leukocyte protease inhibitor to wild-type mice with SCI, sustains improvement in locomotor control and reduces secondary tissue damage, suggesting a protective effect of early neutrophil recruitment after spinal cord contusion (176). Overall, given these conflicting data and the lack of knowledge about the precise role of neutrophils in SCI, further studies are required.

## Extracellular Traps in CNS Autoimmune Diseases

Few data have explored the contribution of DNA fibers released by immune cells to CNS autoimmune pathology. Multiple Sclerosis (MS) is a chronic inflammatory and demyelinating disorder of the CNS that affects about 2 million people worldwide, with a female-to-male ratio of 3:1 (177) and is the leading cause of non-traumatic neurological disability in the young adult population (177). In MS it has been hypothesized that exposure of genetically predisposed individuals to unknown environmental factors (e.g. virus) generates a misdirected autoimmune T cell response against myelin in the CNS, resulting in the formation of multifocal areas of inflammation, extensive demyelination, and neurodegeneration (178). More recently, a crucial contribution to the pathogenesis of MS has also been proposed for Epstein-Barr virus-infected B lymphocytes (179). Although neutrophils do not populate inflammatory CNS lesions in MS, studies in experimental models of CNS autoimmunity such as experimental autoimmune encephalomyelitis (EAE), have proposed that neutrophils could contribute to the early development of lesions (180), promoting BBB disruption at the meningeal interface (181) or stimulating the maturation of CNS-resident antigen-presenting cells (182). In MS patients, plasma levels of elastase correlate with clinical disability and cumulative magnetic resonance imaging lesion volume on T1 weighted sequence, an indicator of tissue damage (180). Serum levels of MPO-DNA complexes in MS are significantly increased compared to healthy donors and gender stratification revealed higher amounts in male MS subjects than females (183). Whether circulating MPO-DNA complexes derive from ETs or other cell death modalities needs further verification. In the priming phase of chronic EAE (i.e. seven days after immunization) induced in C57BL/6 mice with myelin oligodendrocyte glycoprotein peptide 35-55 (MOG_35-55_), we observed marked staining for DNA and CitH3 in proximity to CD4+ cells in the draining lymph nodes, compared to naïve animals (87). CD4+ T cells purified from EAE mice release extracellular DNA threads positive for MitoTracker in a mtROS-dependent way upon re-stimulation with the immunization peptide, but not an irrelevant peptide (87). Interestingly, increased levels of cell-free mtDNA were observed in the serum of EAE mice in comparison to naïve mice. Furthermore, the treatment of EAE mice with a mtROS inhibitor dampened EAE severity and the pro-inflammatory T cell response against myelin, suggesting the involvement of DNA release from CD4+ T cell in CNS autoimmune inflammation (87). However, the therapeutic effect of mtROS could also be related to other cell targets or mechanisms, so further studies are necessary to understand the relevance of this process *in vivo*.

Augmented levels of elastase have also been detected in the plasma of patients with NMO (184), an autoimmune and demyelinating disease that primarily affects the spinal cord and the optic nerve with significant neutrophil infiltration into the CNS (53). NMO is characterized by a Th17, granulocyte, and interferon signature (185). The treatment of neutrophils with C5a and INF-β results in the release of NETs decorated with histone and elastase, suggesting NMO inflammatory milieu is favorable to NETs formation (184). Of note, treatment of a NMO-like disease model with an elastase inhibitor promotes a remarkable clinical recovery and reduction of CNS inflammatory lesions (184).

## Extracellular Traps in Brain Tumors

Gliomas are the most common primary CNS tumors in adults and are classified into varying degrees of increasing malignancy based on histologic and molecular characteristics (186). Approximately 50% of patients develop glioblastoma multiforme (GBM), the most aggressive glioma subtype, characterized by a poor prognostic outcome (187). GBM has been proposed to originate from driver mutations in neural stem cells of the subventricular zone of the adult human brain, but the highly immune-suppressive microenvironment of GBM is key contributor to disease progression (188). Rather than promoting anti-tumor immune responses, NETs support neoplastic growth and metastatic processes by multiple mechanisms (189). Indeed, NETs can provide a physical shield that protects tumor cells from T and NK cell-mediated immune attack (190), or directly support tumor cell proliferation and invasiveness by activating the CCDC25-integrin linked kinase-β-parvin pathway (191) or TGF-β signaling cascade (192). NETs have been shown to promote proliferation and invasiveness of glioma cells *via* HMGB1-mediated stimulation of RAGE and activation of the NF-kB signaling *in vitro* (193). Enhanced brain infiltration of CD66b+ granulocytes with intense staining for CitH3 and MPO has been described in the brain tissue of high-grade gliomas compared to low-grade ones (193). Since CD66b is also a marker of myeloid-derived suppressor cells, highly enriched in the tumor microenvironment (194), it might be worth evaluating whether ETs detected in GBM tissue could originate from this myeloid subpopulation. A recent report identified histone H1-coated DNA fibers colocalizing with microglia in glioblastoma brain tissue, suggesting a possible involvement of microglial ETs in GBM pathology (83).

## Discussion

Despite the enormous amount of work performed so far, the data discussed in this review highlight that still many open questions deserve to be addressed to better understand the biology of ETosis and, more generally, the phenomenon of self-DNA extrusion from immune cells. The concept of ETs release *via* suicidal cell death as an immune defense mechanism against pathogens has been questioned by some authors doubting on why a cell would program self-destruction to fight an incoming microbial attack, rather than proceed to phagocytosis. In parallel, it is not so obvious to figure out that, in the plethora of infectious and noninfectious disorders in which NETs have been implicated, there is a systematic and massive leakage of intracellular components into the extracellular compartment due to suicidal NETosis, which could expose potential cryptic antigens to the immune system, with major challenges to self-tolerance mechanisms. Of note, one work demonstrated that immunization of mice with myeloid dendritic cells loaded with suicidal NETs components induces autoimmune vasculitis and antineutrophil cytoplasmic antibodies (195). The discovery of vital ETs release suggests that secretion of extracellular DNA fibers by neutrophils and other immune cells might occur in more controlled and less pervasive ways than in suicidal NETosis and that the latter modality might be involved in a restricted number of conditions than previously thought, most likely in in chronic inflammatory and autoimmune disorders.

A crucial aspect that needs to be elucidated is why only a small proportion of neutrophils undergo NETosis. In this regard, it might be interesting to verify whether there is a different ability to release NETs between subtypes of neutrophils, such as N1 neutrophils, which are pro-inflammatory or anti-tumorigenic, and N2 neutrophils, which are anti-inflammatory or pro-tumorigenic, or the high-density versus low-density neutrophils (196). The scenario is even more complicated in the case of adaptive immune cells, since the characterization of extracellular DNA threads is still at an early stage and seems to involve both viable (mtDNA) and lytic (histone-coated DNA fibers) extrusion mechanisms. DNA release from stimulated B cells was assessed only after a short incubation period (90), but it might be worthwhile to test whether a lytic form of ETosis in B cells occurs after longer times in culture.

A significant number of studies have indicated ETs involvement in several CNS neurological disorders, based on both clinical findings and evidence from experimental models (summarized in **Table 1**). However, some studies assume the involvement of NETosis in a specific disease by performing immuno-staining in the target tissue with markers such as MPO and elastase, which cannot discriminate between ETs deriving from neutrophils, monocytes or microglial cells. Moreover, the evidence of multiple ETs-releasing cells suggests reconsidering *in vivo* approaches that have utilized DNase or mice deficient in elastase, MPO or PAD4 to demonstrate the contribution of NETs in a specific context. The characterization and potential identification of ETs markers specific to each immune cell subset could provide an important advance in evaluating how ETs derived from different cells contribute to the immunopathology of CNS disorders. In the meantime, at least the proximity of ETs markers to the immune cell that is hypothesized to release ETs should be demonstrated in the target tissue. Finally, a special effort should be directed to detail ETs extrusion mechanisms more specifically for each immune cell subtype. This would be particularly valuable from a therapeutic point of view, because it would allow targeting selective inflammatory components according to the specific CNS pathological context.

**Table 1:** ETs in the pathogenesis of CNS disorders.

Human Disease	Biological Setting	ETs origin	Proposed mechanism of action/evidence	Refs.
AD	Human samples 5xFAD/3xTg-AD mouse models	Neutrophils	Chronic BBB damage and inflammation. Intravascular and parenchymal NETs accumulation.	(99)
ALS	Human samples SOD1G93A rat model	Neutrophils/ Mast cells?	Peripheral motor neuron degeneration and paralysis progression. Degenerating skeletal muscles.	(118)
IS	Human samples	Neutrophils	CitH3+ NETs retrieved in thrombi. CitH3+ MPO+NE+ NETs in brain tissue. NETs as scaffold responsible for platelet adhesion and thrombus resistance to t-PA. NETs content in the cerebral thrombus differs according to stroke etiology. Thrombus NETs content correlates with NETs plasma level at stroke onset.	(133, 134, 136, 137, 143)
	MCAO in WT and *Il1α/β* ^−/−^ mice	Neutrophils	TLR4-driven intravascular inflammation and IL-1-dependent cerebrovascular transmigration of neutrophils	(128)
	MCAO in rat MCAO in HMGB1^fl/fl^ PF4-Cre MCAO in *Padi4* ^−/−^ and *cGas* ^−/−^ mice	Neutrophils Neutrophils	Pathological cross-talk between HMGB1 and NETosis NETs contributes to tPA-induced BBB breakdown *via* cGAS-STING activation and type 1 IFN response	(132, 134, 142)
TBI	Human samples CCI model in WT or *Tlr4* ^-/-^ mice DAI rat model CCI model in WT or *Ptx3* ^-/-^ mice	Neutrophils Neutrophils Neutrophils Neutrophils	NETs formation correlates with elevated ICP and worsening in neurological function. Reduced serum DNase activity and high cell-free DNA. TLR-4- and PAD4-dependent NETs release mediates cerebral hypoperfusion/edema. NETs contribute to secondary sympathetic hyperactivity through LL37-P2X7R microglial activation NET-bound PTX3 in the injured cortex in the acute phase.	(151, 153, 155, 162)
SCI	Clip-compression rat model	Neutrophils	NETs exacerbate secondary fibrotic scar formation and BBB damage through endothelial TRPV4 calcium-permeable channel.	(173)
MS	Human samples MOG_35-55_-induced EAE	Neutrophil CD4+ T lymphocytes	Higher circulating MPO-DNA complexes in RRMS patients *vs* other MS subtypes Gender differences in circulating NETs in RRMS patients CitH3+CD4+ DNA threads in draining lymph nodes during the priming phase of disease	(87, 183)
NMO	Human samples	Neutrophil?	Increased circulating levels of NE	(184)
GBM	Human samples Human samples	Neutrophil Microglial cells	CitH3/MPO+ NETs in high grade vs low grade gliomas. DNA/Histone 1+ ETs in GBM brain tissue	(83, 193)

AD, Alzheimer’s Disease; ALS, Amyotrophic lateral sclerosis; APCs, antigen-presenting-cells; BBB, blood-brain barrier; CCI, controlled cortical impact; CitH3, citrullinated histone H3; DAI, diffuse axonal injury; GBM, Glioblastoma; ICP, intracranial pressure; IFN, interferon; IS, ischemic stroke; MCAO, middle cerebral artery occlusion; MOG_35-55_, myelin oligodendrocyte glycoprotein peptide 35-55; MPO, myeloperoxidase; MS, multiple sclerosis; NE, neutrophil elastase; NETs, neutrophil extracellular traps; NMO, neuromyelitis optica; PTX3, Pentraxin 3; RRMS, relapsing-remitting multiple sclerosis; Refs, references; SCI, spinal cord injury; t-PA, tissue Plasminogen Activator; TBI, traumatic brain injury; TLR-4, toll-like receptor 4; TRPV4, transient receptor potential vanilloid type 4; WT, wild-type.

## Author Contributions

Authors declare that they have substantially participated in the preparation and writing of the manuscript and have taken due care regarding their contribution to ensure the integrity of the work. All authors contributed to the article and approved the submitted version.

## Funding

This work was supported by FISM - Fondazione Italiana Sclerosi Multipla – cod. 2019/R-Single/065 and financed or co-financed with the ‘5 per mille’ public funding, and partially supported by the Italian Ministry of Health (RRC).

## Conflict of Interest

The authors declare that the research was conducted in the absence of any commercial or financial relationships that could be construed as a potential conflict of interest.

## Publisher’s Note

All claims expressed in this article are solely those of the authors and do not necessarily represent those of their affiliated organizations, or those of the publisher, the editors and the reviewers. Any product that may be evaluated in this article, or claim that may be made by its manufacturer, is not guaranteed or endorsed by the publisher.

## Abbreviations

AD, Alzheimer’s disease; Ab, antibody; BBB, blood-brain barrier; CCI, controlled cortical impact; CNS, central nervous system; CitH3, citrullinated histone H3; cGAS, cyclic GMP-AMP;cGAMP) synthase; CR, complement receptor; CXCL, C-X-C motif chemokine ligand; CXCR, C-X-C chemokine receptor; DAMPs, damage-associated molecular patterns; ECP, eosinophil cationic protein; ETs, extracellular traps; GBM, glioblastoma multiforme; IL, interleukin; IFN, interferon; ICP, intracranial pressure; IS, ischemic stroke; LPS, lipopolysaccharide; MLKL, mixed lineage kinase domain-like; MBP, major basic protein; MMP, matrix metalloproteinase; MCAO, middle cerebral artery occlusion; mt, mitochondrial; MN, motor neuron; MPO, myeloperoxidase; NE, neutrophil elastase; NETs, neutrophil extracellular traps; NADPH, nicotinamide adenine dinucleotide phosphate; NLR, NOD-like receptor; NLRP3, NLR family pyrin domain containing 3; PAD, peptidyl-arginine deiminase; PAF, platelet-activating factor; PD, Parkinson’s disease; PMA, phorbol myristate acetate; PPAR, peroxisome proliferator-activated receptor; RAGE, receptor for advanced glycation endproducts; RIPK, receptor-interacting protein kinase; RNP, ribonucleoprotein; ROS, reactive oxygen species; SCI, spinal cord injury; t-PA, tissue plasminogen activator; TLR, toll-like receptor; TFAM, transcription factor A mitochondria; TRPV4, transient receptor potential vanilloid type 4; TBI, traumatic brain injury; TNF, tumor necrosis factor. A mitochondria; TRPV4, transient receptor potential vanilloid type 4; TBI, traumatic brain injury; TNF, tumor necrosis factor.
